# DFT Study of Au_3_In and Au_3_In_2_ Intermetallic Compounds: Structural Stability, Fracture Toughness, Anisotropic Elasticity, and Thermophysical Properties for Advanced Applications

**DOI:** 10.3390/ma18071561

**Published:** 2025-03-29

**Authors:** Ching-Feng Yu, Yang-Lun Liu

**Affiliations:** 1 Department of Mechanical Engineering, National United University, Miaoli 360303, Taiwan; 2 Department of Aerospace and Systems Engineering, Feng Chia University, Taichung 40724, Taiwan

**Keywords:** density functional theory, intermetallic compounds, microelectronic packaging technologies, mechanical properties, thermophysical properties

## Abstract

This study systematically explores the structural stability, mechanical properties, elastic anisotropy, fracture toughness, and thermophysical characteristics of Au_3_In and Au_3_In_2_ intermetallic compounds (IMCs) through density functional theory (DFT) simulations. Employing the generalized gradient approximation (GGA) and the Voigt–Reuss–Hill approximation enables precise predictions of polycrystalline elastic behavior, providing critical insights into the intrinsic stability and mechanical anisotropy of these IMCs. Structural optimization identifies the equilibrium lattice parameters and cohesive energies, indicating stronger atomic bonding and superior structural stability in Au_3_In relative to Au_3_In_2_. Elastic constant calculations confirm mechanical stability and reveal pronounced anisotropic elastic behavior; Au_3_In exhibits significant stiffness along the [010] crystallographic direction, while Au_3_In_2_ demonstrates notable stiffness predominantly along the [001] direction. Both Au_3_In and Au_3_In_2_ exhibit ductile characteristics, confirmed by positive Cauchy pressures and elevated bulk-to-shear modulus (*K*/*G*) ratios. Fracture toughness analysis further establishes that Au_3_In offers greater resistance to crack propagation compared to Au_3_In_2_, suggesting its suitability in mechanically demanding applications. Thermophysical property evaluations demonstrate that Au_3_In possesses higher thermal conductivity, elevated Debye temperature, and superior volumetric heat capacity relative to Au_3_In_2_, reflecting its enhanced capability for effective thermal management in electronic packaging. Anisotropy assessments, utilizing both universal and Zener anisotropy indices, reveal significantly higher mechanical anisotropy in Au_3_In_2_, influencing its practical applicability.

## 1. Introduction

In recent years, the study of the physical properties of intermetallic compounds (IMCs) has emerged as a central area of focus in the field of materials engineering [1,2,3,4,5]. This surge in interest is largely attributed to the pivotal role these materials play in influencing the efficiency, reliability, and performance of electronic packaging interconnections. IMCs are distinguished by their remarkable magnetic, superconducting, and chemical properties, which are rooted in their robust internal structures and metallic bonding. Among the various IMCs, the gold–indium (Au-In) system has garnered substantial attention, particularly for its relevance in microelectronic packaging applications. Within this system, the Au_3_In and Au_3_In_2_ phases have been extensively studied experimentally due to their significant implications for both practical applications and fundamental scientific understanding. However, current literature primarily focuses on experimental investigations, with limited computational studies such as density functional theory (DFT) or ab initio methods reported thus far.

The interfacial interactions between gold and indium are critical to the formation of these phases, making them an area of considerable research interest. The unique properties of Au_3_In and Au_3_In_2_ have been the subject of numerous experimental investigations, particularly regarding their advantageous roles in electronic device packaging. Klumel et al. [6] conducted a comprehensive experimental analysis of the synthesis and application of Au_3_In, highlighting its significance in improving the reliability and fatigue performance of solder joints in high-power diode lasers. According to their results, the formation of Au-In intermetallic compounds, including Au_3_In, occurs during soldering processes under elevated temperatures exceeding the melting point of indium (156 °C), leading to the rapid formation of these compounds even at moderate thermal conditions ranging from 40 to 140 °C. Otnes et al. [7] experimentally studied the formation of Au_3_In to address challenges in pattern preservation during the growth of indium phosphide nanowires. They implemented a pre-anneal nucleation step at approximately 300 °C, significantly enhancing pattern preservation. Jany et al. [8] performed experiments involving the deposition of gold onto InAs (001) surfaces under ultra-high vacuum conditions, resulting in crystalline Au_3_In nanostructures, characterized through techniques such as SEM, AFM, and HAADF-STEM. Ivey et al. [9] examined experimentally the fabrication of ohmic contacts using Au-based metallizations on InP substrates, highlighting various intermetallic stages during annealing. Eyring et al. [10] employed high-resolution electron microscopy to experimentally study the dynamic formation process of Au_3_In by depositing gold islands onto an indium film.

**Figure 1:**
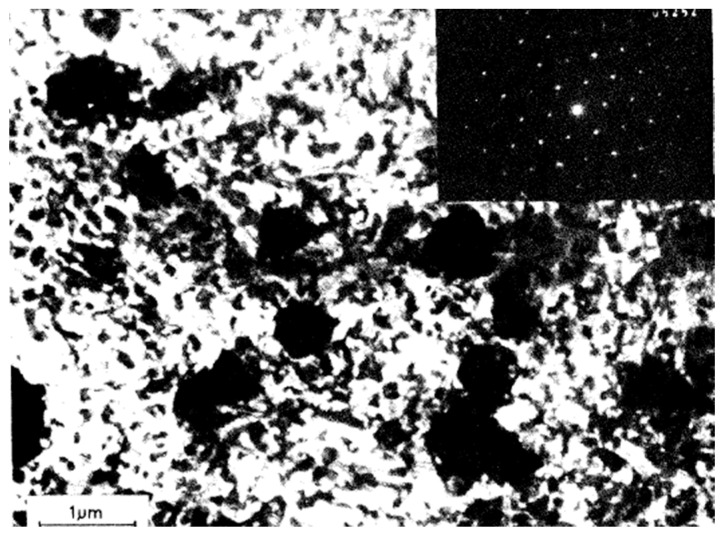
Bright-field TEM micrograph illustrating the Au/InP interface following annealing at 360 °C for 60 min. The inset displays a transmission electron diffraction pattern corresponding to the Au_3_In phase [12].

Similarly, Goral et al. [11] used electron microscopy to experimentally analyze the growth of Au_3_In IMC in gold–indium diffusion couples. Piotrowska et al. [12] conducted experimental studies using XRD, TEM, and SEM to investigate binary compound formation in Au/InP systems under thermal annealing. They observed the presence of the Au_3_In phase (Figure 1). Weizer et al. [13] and Clausen et al. [14] also provided experimental insights into the formation and optimization of Au_3_In in ohmic contacts on InP substrates through thermal annealing. Swenson et al. experimentally examined phase equilibria at 600 °C, confirming the thermodynamic stability of Au_3_In via XRD analysis. Wada et al. [15] experimentally studied the kinetics of Au_3_In formation through interdiffusion processes between gold and indium films, reporting an activation energy of 2.31 eV.

**Figure 2:**
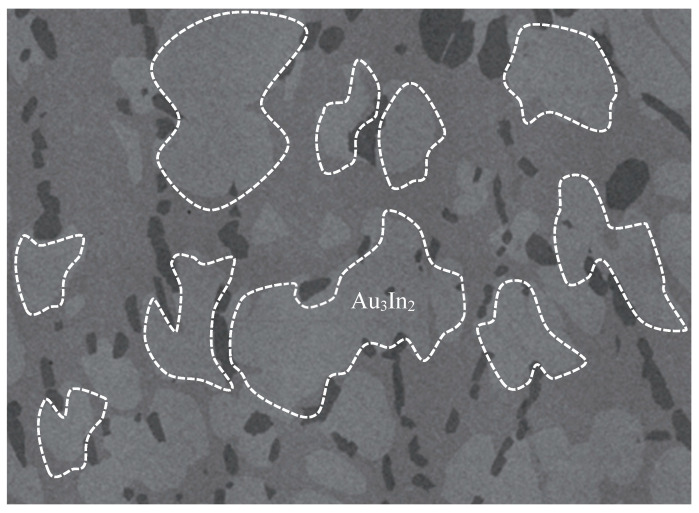
SEM micrograph of Au_3_In_2_ [22].

The Au_3_In_2_ phase has also attracted considerable experimental research interest due to its utility in forming ohmic contacts on III-V compound semiconductors, such as indium phosphide and gallium arsenide. Experimental studies [8,11] demonstrated that rapid thermal annealing facilitates the emergence of this phase, along with related IMCs. Huang et al. [16] experimentally demonstrated that Au_3_In_2_ forms during rapid thermal annealing involving Au/Si/Ni ohmic contacts on InP substrates, significantly reducing specific resistance. Cherneva et al. [17] explored experimentally the mechanical properties of electrochemically deposited Au-In alloy films containing Au_3_In_2_ through nano-indentation techniques. Wodniecki et al. [18] experimentally investigated quadrupole interactions in Au_3_In_2_ using perturbed angular correlation methods, revealing temperature-dependent transformations. Wei et al. [19] experimentally studied the formation of indium nanohillocks mediated by Au_3_In_2_ layers, emphasizing their role in modulating surface properties. Póczi et al. [20] identified the formation of Au_3_In_2_ experimentally during vacuum annealing of gold-coated InP systems. Vandenberg et al. [21] experimentally assessed the thermal stability of Au_3_In_2_ in Au/InGaAs/InP systems, observing phase formation at temperatures as low as 160 °C. Ghasemi et al. [22] conducted a systematic experimental study of Au_3_In_2_ phase equilibria employing DTA, XRD, EDS, and SEM techniques. Figure 2 illustrates the experimentally observed morphology of Au_3_In_2_ [22].

The literature reviewed above indicates a predominant focus on experimental studies for both Au_3_In and Au_3_In_2_ intermetallic compounds, revealing a significant gap in computational approaches such as DFT or ab initio calculations, which remain relatively unexplored for these IMC systems. Despite extensive investigations into these materials, a truly comprehensive understanding of their mechanical and thermal properties remains limited, with many issues still open regarding how their fundamental properties can be effectively leveraged in practical applications. To the best of the authors’ knowledge, only the hardness of Au_3_In_2_ has previously been investigated [17].

Using first-principles calculations grounded in DFT, this study investigates the crystallographic, elastic, and thermophysical characteristics of orthorhombic Au_3_In and trigonal Au_3_In_2_. The simulations are performed within the pseudopotential framework, employing the generalized gradient approximation (GGA) to account for exchange–correlation effects [23,24,25]. The mechanical properties are analyzed using the Voigt–Reuss–Hill approximation [26] to determine the effective polycrystalline elastic behavior, providing a comprehensive assessment of the mechanical stability and anisotropy of these intermetallic compounds. The validity of the theoretical models is established through comparisons with available experimental data [17,27,28,29,30]. The investigation of mechanical properties includes elastic moduli, anisotropy, brittleness, ductility, fracture toughness, and microhardness, all of which are essential for evaluating the structural integrity and potential applications of these materials in solder interconnects. Thermophysical analyses focus on Debye temperature, heat capacity, coefficients of thermal expansion (CTE), and thermal conductivities, encompassing both lattice thermal conductivity and minimum thermal conductivity. Additionally, the study examines the directional dependence of elastic properties, offering insights into the anisotropic mechanical behavior of Au_3_In and Au_3_In_2_.

## 2. Methods

This study employed a rigorous and comprehensive ab initio investigation grounded in the principles of DFT [23,24] to systematically evaluate and characterize the structural, electronic, and thermodynamic properties of the Au_3_In and Au_3_In_2_ IMCs. The computational framework utilized for this analysis was the well-established Cambridge Serial Total Energy Package (CASTEP) [31], a plane-wave-based DFT code renowned for its accuracy and robustness in modeling the electronic structure and physical properties of complex materials. CASTEP has been extensively validated and widely adopted in the scientific community for the in-depth investigation of various metallic systems and intermetallic compounds, as evidenced by numerous prior studies [32,33,34].

Within the CASTEP framework, the ultrasoft pseudopotential method [35] was employed to accurately describe the intricate interactions between ionic cores and valence electrons. This approach effectively reduces computational complexity while maintaining high accuracy by replacing strongly oscillating all-electron wavefunctions with computationally efficient pseudo-wavefunctions. The exchange–correlation energy, a fundamental component of the total energy functional in DFT, was treated using the Perdew–Burke–Ernzerhof (PBE) formulation of the generalized gradient approximation (GGA) [25]. The PBE-GGA functional was selected due to its well-documented balance between computational efficiency and predictive accuracy, particularly for intermetallic compounds.

The ground-state electronic configurations and optimized crystal structures of Au_3_In and Au_3_In_2_ were determined through an iterative energy minimization procedure implemented via the Broyden–Fletcher–Goldfarb–Shanno (BFGS) quasi-Newton algorithm [36]. This optimization algorithm is known for its robust convergence properties and efficiency in identifying the lowest-energy configuration of a system. For the pseudo-atomic calculations, the valence electron configurations were explicitly defined as [5d^10^6s^1^] for Au and [5s^2^5p^1^] for In, ensuring an accurate representation of their respective electronic structures.

To ensure numerical precision and convergence, the plane-wave basis set cut-off energy was carefully selected and fixed at 440 eV for all calculations. This value was determined based on rigorous convergence tests to achieve an optimal trade-off between computational accuracy and efficiency. Furthermore, Brillouin-zone [37] sampling, which is critical for accurately integrating electronic states in reciprocal space, was performed using a systematically generated Monkhorst–Pack *k*-point mesh [38]. Specifically, an 8 × 10 × 10 mesh was employed for Au_3_In, while a denser 12 × 12 × 8 mesh was used for Au_3_In_2_, ensuring uniform and adequate sampling of the Brillouin zone.

During the geometry optimization phase, highly stringent convergence criteria were applied to ensure the highest level of accuracy and reliability in the computed structural parameters. The maximum energy change per atom, maximum force acting on each atom, maximum stress tensor component, and maximum ionic displacement were set to highly conservative thresholds of 5 × 10^−6^ eV/atom, 0.01 eV/Å, 0.02 GPa, and 5 × 10^−4^ Å, respectively. These stringent criteria were imposed to guarantee well-converged and physically meaningful results, which are essential for subsequent analyses of the structural stability and thermodynamic properties. By employing this rigorous and systematically validated computational methodology, this study ensures the accuracy and reliability of the derived physical properties of Au_3_In and Au_3_In_2_. The insights obtained from this investigation provide a fundamental basis for understanding the behavior of these intermetallic compounds and their potential applications, particularly in thermal management and electronic packaging technologies.

## 3. Results

### 3.1. Structural Properties and Phase Stability

**Figure 3:**
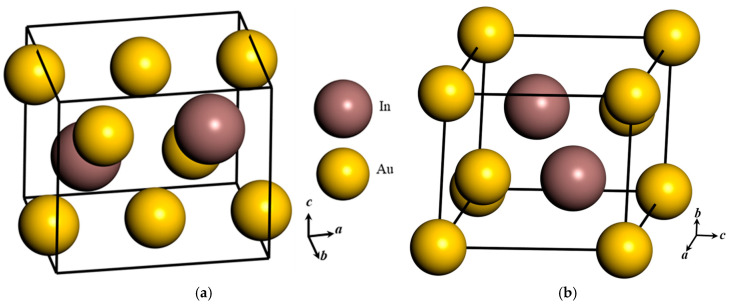
Crystal structure of (**a**) orthorhombic Au_3_In unit cell and (**b**) trigonal Au_3_In_2_ unit cell.

The orthorhombic Au_3_In crystal structure is classified within the Pmmn space group, while the trigonal Au_3_In_2_ crystal belongs to the P-3m1 space group. These crystallographic designations are fundamental to understanding the symmetry constraints and atomic arrangements that govern the structural and electronic properties of these crystals. The reduced atomic coordinates of Au and In atoms within the Au_3_In and Au_3_In_2_ crystals are systematically tabulated in Table 1 and Table 2 [27,28], providing a detailed representation of their atomic configurations. Additionally, the corresponding crystal structures are depicted in Figure 3, offering a visual interpretation of atomic spatial distributions within the unit cells. To achieve structural equilibrium, optimization was performed based on experimentally determined crystal structures obtained via X-ray diffraction (XRD) analysis [27,28]. This computational optimization process yielded the equilibrium lattice parameters and relaxed atomic configurations for the Au_3_In and Au_3_In_2_ unit cells at their lowest energy states, ensuring consistency between theoretical predictions and experimentally observed crystallographic features [27,28,29,30]. The refined reduced atomic coordinates for Au_3_In and Au_3_In_2_, as presented in Table 3 and Table 4, further elucidate the stability of reduced atomic positions within these structures. The lattice constants for Au_3_In were determined to be 5.907, 4.763, and 5.210 Å, whereas those for Au_3_In_2_ were 4.635, 4.635, and 5.691 Å, as summarized in Table 5. These lattice parameters play a crucial role in defining the geometric configuration of each crystal system. Moreover, the equilibrium unit cell volumes for Au_3_In and Au_3_In_2_ were computed to be 146.57 Å^3^ and 105.89 Å^3^, respectively, offering insights into the volumetric characteristics and density variations between the two compounds.

A comparative analysis of the calculated structural parameters with experimentally reported values reveals a strong agreement, with deviations in lattice constants and unit cell volumes observed within a narrow range. For Au_3_In, the discrepancies in lattice parameters fall between 0.36% and 1.17%, while deviations in unit cell volumes range from 0.49% to 1.91%. Similarly, for Au_3_In_2_, the differences in lattice constants are within 0.55% to 2.16%, whereas the variations in unit cell volumes extend from 4.33% to 4.97%. Although minor discrepancies are observed, they remain within an acceptable range, indicating the robustness and reliability of the computational methodology employed. The reasonable agreement between the theoretically optimized lattice parameters and experimentally measured values underscores the validity of the adopted structural optimization approach, reinforcing its efficacy in accurately capturing the fundamental crystallographic characteristics of Au_3_In and Au_3_In_2_.

In order to further assess the structural stability of the Au-In IMCs, both the cohesive energy (*E_c_*) and the formation enthalpy (Δ*H*) were computed. The cohesive energy represents the energy required to dissociate the compound into its constituent isolated atoms and thus serves as an important indicator of structural stability. Generally, a lower cohesive energy corresponds to a more stable structure. The cohesive energy of Au_3_In and Au_3_In_2_ IMCs can be determined using the following equation:

(1) $$E_{c}=\left[E_{total}-\left(xE_{Au}+yE_{In}\right)\right]/\left(x+y\right),$$

where *E_total_* is the total energy of the alloy at its equilibrium lattice parameters, *E_Au_* and *E_In_* denote the energies of isolated Au and In atoms, respectively, and *x* and *y* represent the atomic numbers of Au and In in the unit cell. According to the calculated results summarized in Table 5, the cohesive energies for Au_3_In and Au_3_In_2_ are −3.183 eV/atom and −3.119 eV/atom, respectively. Since Au_3_In exhibits a more negative cohesive energy than Au_3_In_2_, it indicates stronger atomic bonding and a lower tendency for decomposition, thereby suggesting that Au_3_In is structurally more stable than Au_3_In_2_.

The formation enthalpy (Δ*H*) provides insight into the thermodynamic favorability of the compound by quantifying the energy released or absorbed when the compound forms from its constituent elements. A more negative value of Δ*H* generally indicates a greater propensity for compound formation and, consequently, higher stability. The formation enthalpy of Au_3_In and Au_3_In_2_ IMCs were evaluated using the following equation:

(2) $$\Delta H=\left[E_{total}-\left(xE_{Au}^{bulk}+yE_{In}^{bulk}\right)\right]/\left(x+y\right),$$

$E_{Au}^{bulk}$ and $E_{In}^{bulk}$ correspond to the atomic energies of a single Au and In atom in their bulk states, respectively. Table 5 presents the calculated formation enthalpy of Au_3_In and Au_3_In_2_, with values of −17.639 kJ/mol and −14.399 kJ/mol, respectively. The more negative formation enthalpy of Au_3_In suggests that it is more thermodynamically stable than Au_3_In_2_, indicating a stronger tendency for its formation.

Overall, the comparison of cohesive energy and formation enthalpy between Au_3_In and Au_3_In_2_ reveals a consistent trend in their relative stability. The more negative cohesive energy of Au_3_In (−3.183 eV/atom) compared to Au_3_In_2_ (−3.119 eV/atom) indicates that Au_3_In exhibits stronger atomic bonding, leading to a more structurally stable phase. Similarly, the formation enthalpy of Au_3_In (−17.639 kJ/mol) is more negative than that of Au_3_In_2_ (−14.399 kJ/mol), suggesting that Au_3_In has a greater thermodynamic driving force for formation. These results collectively indicate that Au_3_In is both structurally and thermodynamically more stable than Au_3_In_2_, making it the more favorable intermetallic compound.

### 3.2. Elastic and Mechanical Properties

#### 3.2.1. Phase Stability

The elastic coefficients are intrinsically linked to both the microscopic and macroscopic physical properties of materials, providing critical insights into their structural stability and stiffness. These coefficients serve as fundamental indicators of a material mechanical behavior, revealing essential information about its resilience under various stress conditions. Furthermore, the elastic constants can be quantitatively determined through the generalized formulation of Hooke’s law, allowing for a rigorous evaluation of the mechanical stability and deformation characteristics of a material. This approach provides a comprehensive framework for analyzing how a material responds to various stress conditions, ensuring a more precise assessment of its behavior under load. The generalized formulation of Hooke’s law, which is outlined below, serves as a crucial tool in predicting and quantifying the elastic response of materials to external forces.

(3) $$\sigma_{ij}=C_{ijkl}\varepsilon_{kl},$$

The Cauchy stress tensor, denoted as *σ_ij_*, and the infinitesimal strain tensor, represented as *ε_kl_*, correspond to the stress and strain tensors, respectively. The elastic stiffness tensor is denoted as *C_ijkl_*. It is often beneficial to use matrix notation, known as Voigt notation, to express Hooke’s law. By leveraging the symmetry in the stress and strain tensors, they can be represented as six-dimensional vectors in an orthonormal coordinate system (e_1_, e_2_, e_3_), as follows:

(4) $$[\sigma ]=\left[\begin{array}{c} \begin{array}{c} \begin{array}{c} \sigma_{11}\\ \sigma_{22}\\ \sigma_{33}\\ \sigma_{23}\\ \sigma_{13}\\ \sigma_{12} \end{array} \end{array} \end{array}\right]=\left[\begin{array}{c} \begin{array}{c} \begin{array}{c} \sigma_{1}\\ \sigma_{2}\\ \sigma_{3}\\ \sigma_{4}\\ \sigma_{5}\\ \sigma_{6} \end{array} \end{array} \end{array}\right];\;[\varepsilon ]=\left[\begin{array}{c} \begin{array}{c} \begin{array}{c} \varepsilon_{11}\\ \varepsilon_{22}\\ \varepsilon_{33}\\ 2\varepsilon_{23}\\ 2\varepsilon_{13}\\ 2\varepsilon_{12} \end{array} \end{array} \end{array}\right]=\left[\begin{array}{c} \begin{array}{c} \begin{array}{c} \varepsilon_{1}\\ \varepsilon_{2}\\ \varepsilon_{3}\\ \varepsilon_{4}\\ \varepsilon_{5}\\ \varepsilon_{6} \end{array} \end{array} \end{array}\right],$$

Then, the stiffness tensor can be expressed as

(5) $$[C]=\left[\begin{array}{cccccc} C_{1111} & C_{1122} & C_{1133} & C_{1123} & C_{1131} & C_{1112}\\ C_{2211} & C_{2222} & C_{2233} & C_{2223} & C_{2231} & C_{2212}\\ C_{3311} & C_{3322} & C_{3333} & C_{3323} & C_{3331} & C_{3312}\\ C_{2311} & C_{2322} & C_{2333} & C_{2323} & C_{2331} & C_{2312}\\ C_{3111} & C_{3122} & C_{3133} & C_{3123} & C_{3131} & C_{3112}\\ C_{1211} & C_{1222} & C_{1233} & C_{1223} & C_{1231} & C_{1212} \end{array}\right]=\left[\begin{array}{cccccc} C_{11} & C_{12} & C_{13} & C_{14} & C_{15} & C_{16}\\ C_{12} & C_{22} & C_{23} & C_{24} & C_{25} & C_{26}\\ C_{13} & C_{23} & C_{33} & C_{34} & C_{35} & C_{36}\\ C_{14} & C_{24} & C_{34} & C_{44} & C_{45} & C_{46}\\ C_{15} & C_{25} & C_{35} & C_{45} & C_{55} & C_{56}\\ C_{16} & C_{26} & C_{36} & C_{46} & C_{56} & C_{66} \end{array}\right],$$

and Hooke’s law is written as

(6) $$\left\{\sigma_{i}\right\}=[C_{ij}]\left\{\varepsilon_{i}\right\},$$

For the orthorhombic Au_3_In crystal, the stiffness matrix consists of nine independent elastic constants, as shown below [39]:

(7) $$[C_{ij}]=\left[\begin{array}{cccccc} C_{11} & C_{12} & C_{13} & 0 & 0 & 0\\ C_{21} & C_{22} & C_{23} & 0 & 0 & 0\\ C_{31} & C_{32} & C_{33} & 0 & 0 & 0\\ 0 & 0 & 0 & C_{44} & 0 & 0\\ 0 & 0 & 0 & 0 & C_{55} & 0\\ 0 & 0 & 0 & 0 & 0 & C_{66} \end{array}\right],$$

In contrast, the stiffness matrix for the trigonal Au_3_In_2_ crystal includes six independent elastic constants [40]:

(8) $$[C_{ij}]=\left[\begin{array}{cccccc} C_{11} & C_{12} & C_{13} & C_{14} & 0 & 0\\ C_{21} & C_{11} & C_{13} & -C_{14} & 0 & 0\\ C_{31} & C_{32} & C_{33} & 0 & 0 & 0\\ C_{41} & -C_{14} & 0 & C_{44} & 0 & 0\\ 0 & 0 & 0 & 0 & C_{44} & C_{14}\\ 0 & 0 & 0 & 0 & C_{14} & \frac{C_{11}-C_{12}}{2} \end{array}\right],$$

Once the stiffness matrix is determined, the compliance matrix [*S_ij_*], which is the inverse of the stiffness matrix [*C_ij_*], can be calculated. In this study, the elastic constants were obtained by performing linear fitting with strain values of ± 0.001 and ± 0.003. It is noticed that the calculated elastic constants for the crystal must be positive and should satisfy the following Born stability criteria [41].

For the orthorhombic Au_3_In crystal:

(9) $$C_{11}+C_{12}+C_{33}+2C_{12}+2C_{13}+2C_{23}>0,$$

(10) $$C_{11}+C_{22}>2C_{12},\;C_{22}+C_{33}>2C_{23},\;C_{11}+C_{33}>2C_{13},$$

(11) $$C_{ii}>0\;(i=1\sim 6),$$

For the trigonal Au_3_In_2_ crystal:

(12) $$C_{11}-\left|C_{12}\right|>0,\;(C_{11}+C_{12})C_{33}-2C_{13}^{2}>0,\;(C_{11}-C_{12})C_{44}-2C_{44}^{2}>0,$$

The elastic constants of Au_3_In and Au_3_In_2_ IMCs are presented in Table 6. By substituting these calculated values into the corresponding equations (i.e., Equations (9)–(12)), it is demonstrated that all mechanical stability criteria are satisfied. This confirms that the Au_3_In and Au_3_In_2_ single crystals form an intrinsically stable system, further supporting the validity of the present theoretical calculations. The elastic constants *C*_11_, *C*_22_, and *C*_33_ represent the material resistance to linear compressions along the primary crystallographic directions [100], [010], and [001], respectively. These constants provide fundamental insights into the material response to unidirectional stress when compressive forces are applied along specific crystallographic axes.

#### 3.2.2. Comparison of Elastic Constants

For orthorhombic Au_3_In, the value of *C*_22_ = 192.7 GPa is greater than *C*_33_ = 173.1 GPa and *C*_11_ = 160.0 GPa, indicating the highest stiffness along the [010] direction. This suggests that the bonding strength and compressibility are strongest along the [010] axis, followed by the [001] and [100] directions. The elevated stiffness along the [010] direction reflects enhanced atomic interactions and higher resistance to compressive deformation in this axis, showcasing the pronounced anisotropy of Au_3_In. The pronounced bonding strength along the [010] direction implies greater atomic interaction and higher resistance to deformation under compressive loading along this axis. This finding reveals the anisotropic mechanical properties of Au_3_In, wherein the elastic response significantly varies depending on the direction of the applied stress.

On the other hand, for trigonal Au_3_In_2_, *C*_33_ = 169.5 GPa exceeds *C*_11_ = 134.8 GPa, indicating that the bonding strength and compressibility are higher along the [001] direction compared to the [100] direction. Although the anisotropy in Au_3_In_2_ is less pronounced than in Au_3_In, directional dependence of mechanical properties is still evident. This comparison shows the complex mechanical behavior of Au_3_In and Au_3_In_2_, where crystallographic orientation plays a critical role in their elastic response to external stresses.

Beyond the axial elastic constants, the shear elastic constants *C*_44_, *C*_55_, and *C*_66_ provide important insights into the material resistance to shear deformation across different crystallographic planes and directions. The resistance to shear deformation with respect to a tangential stress applied to the (100) plane in the [010] direction of the compound is represented by the elastic constant *C*_44_. Similarly, *C*_55_ measures the resistance to shear deformation on the (010) plane when stress is applied in the [001] direction, illustrating the material sensitivity to shear forces in this crystallographic orientation. Finally, *C*_66_ represents the resistance to shear on the (001) plane when shear stress is applied along the [100] direction.

For both Au_3_In and Au_3_In_2_, the shear elastic constants *C*_44_, *C*_55_, and *C*_66_ are consistently lower than the corresponding axial constants *C*_11_, *C_2_*_2_, and *C*_33_. This suggests that both IMCs are more susceptible to shear deformation than to compressive stress along the principal crystallographic directions. This behavior implies that Au_3_In and Au_3_In_2_ are more easily deformed by shear forces, providing a clearer understanding of how they respond to different mechanical loads. The lower shear elastic constants indicate that the internal structure of the material offers less resistance to shear stresses, making it more prone to deformation under forces acting parallel to the crystallographic planes. This property is essential for evaluating the performance of these materials in applications where shear forces are critical to their structural integrity.

For Au_3_In, the shear elastic constants are observed to follow a specific relationship: *C*_55_ is greater than *C*_44_, which is greater than *C*_66_. This sequence indicates that the material exhibits the highest resistance to shear deformation along the [001] direction on the (010) plane, while the lowest resistance occurs along the [100] direction on the (001) plane. This variation in shear resistance reflects the anisotropic behavior of Au_3_In, driven by its internal atomic arrangement, which affects the material’s ability to resist deformation differently along various directions. Similarly, for Au_3_In_2_, the shear elastic constants exhibit the relationship *C*_44_ greater than *C*_66_, which signifies that the material resists shear deformation more effectively along the [010] direction on the (100) plane compared to the [100] direction on the (001) plane. This consistent trend further underlines the influence of the atomic structure on directional shear properties in Au_3_In and Au_3_In_2_, revealing their anisotropic mechanical response.

#### 3.2.3. Cauchy Pressure

The concept of Cauchy pressure [42] offers valuable insights into the mechanical behavior of materials, particularly in distinguishing between ductile and brittle characteristics. A ductile material is typically characterized by positive Cauchy pressure, whereas a brittle material exhibits negative Cauchy pressure. Cauchy pressure serves as an important indicator for describing the angular nature of atomic bonding within a material. Positive values of Cauchy pressure generally indicate ionic bonding, while negative values are associated with covalent bonding, reflecting the distinct bonding mechanisms that influence the mechanical behavior of materials. This differentiation between ionic and covalent bonding, as elucidated through Cauchy pressure, provides significant insights into the structural and mechanical properties of materials.

For the orthorhombic Au_3_In crystal, Cauchy pressure can be defined as *C*_23_−*C*_44_ for the (100) plane, *C*_13_−*C*_55_ for the (010) plane, and *C*_12_−*C*_66_ for the (001) plane [43]. For the trigonal Au_3_In_2_ crystal, the difference *C*_13_−*C*_44_ is referred to as Cauchy pressure for the (100) and (010) planes, and *C*_12_−*C*_66_ for the (001) plane [44]. The values were found to be 91.0 GPa for the (100) plane, 90.2 GPa for the (010) plane, and 108.4 GPa for the (001) plane, confirming the ductile nature of the orthorhombic Au^3^In crystal due to the positive Cauchy pressure. For the Au_3_In_2_ crystal, the calculated value of *C*_13_−*C*_44_ is 47.0 GPa, which also indicates a positive Cauchy pressure, thereby revealing the ductile characteristics of the Au_3_In_2_ crystal.

A comparison between the Cauchy pressures of Au_3_In and Au_3_In_2_ reveals that both crystals exhibit positive values, indicative of their ductile nature. However, the Cauchy pressures of orthorhombic Au_3_In are notably higher than that of trigonal Au_3_In_2_, with values ranging from 90.2 GPa to ~108.4 GPa for different planes, compared to 47.0 GPa for Au_3_In_2_. This indicates that the orthorhombic Au_3_In exhibits a relatively greater degree of ductility, which may be attributed to differences in crystal structure and atomic bonding characteristics. The higher Cauchy pressures in Au_3_In imply stronger ionic bonding contributions, which enhance ductility compared to the relatively lower value observed for Au_3_In_2_. These differences in Cauchy pressure indicate the impact of crystal symmetry and atomic arrangement on the mechanical properties of these intermetallic compounds.

#### 3.2.4. Kleinman Parameter

The Kleinman parameter, denoted as ζ, is a dimensionless quantity that provides a significant framework for analyzing the mechanical response of crystalline materials to externally applied stresses. The Kleinman parameter of a compound can be estimated using following relation [45]:

(13) $$\zeta =\frac{C_{11}+8C_{12}}{7C_{11}+2C_{12}}.$$

With values constrained within the range of zero to one (0 ≤ ζ ≤ 1), this parameter serves as a critical indicator of the relative contributions of bond deformation mechanisms, specifically bond bending and bond stretching, in the material resistance to external forces. The interpretation of this parameter allows for a deeper understanding of how atomic interactions govern the structural stability of the material under stress. At the lower limit, where the Kleinman parameter ζ = 0, the material resistance to external stress is primarily governed by bond stretching and contracting mechanisms, with bond bending playing a minimal role. This suggests that in such materials, atomic bonds extend or contract along specific crystallographic directions in response to applied forces. The material stiffness and mechanical behavior are largely determined by the strength of direct atomic bonds along these axes. When bond stretching dominates, the material response to stress results in axial shifts in atomic positions, leading to either elongation or compression, depending on the direction of the applied force. These deformation modes are typical in materials where bond stiffness is a key factor in mechanical resilience, and where altering bond lengths requires more energy than other deformation mechanisms.

Conversely, at the upper limit, where the Kleinman parameter ζ = 1, the material mechanical resistance is dominated by bond bending mechanisms. Here, bond stretching and contracting contribute minimally to resisting applied forces. Instead, the material response is largely governed by angular changes between atomic bonds, indicating a more flexible bonding network that allows significant distortion without substantial changes in bond length. This behavior is characteristic of materials where the bonding structure accommodates tilt or rotational movements of atomic planes relative to each other, without high energy costs from altering bond lengths. These materials have greater angular bond flexibility, with bond bending being a crucial factor in defining mechanical properties under stress. For the orthorhombic Au_3_In crystal, the calculated Kleinman parameter ζ = 0.893 indicates that the mechanical response of the material is significantly influenced by bond bending mechanisms, with a moderate contribution from bond stretching. A value of ζ closer to one suggests that angular adjustments in atomic bonds play a crucial role in resisting external stress, allowing the material to accommodate deformation through bond angle flexibility while maintaining structural stability. In contrast, the Kleinman parameter for the trigonal Au_3_In_2_ crystal is ζ = 0.794, indicating that bond bending mechanisms are also present but to a lesser extent than in Au_3_In. The lower ζ value reflects a comparatively stronger contribution from bond stretching in resisting mechanical stress. This suggests that the atomic structure for Au_3_In and Au_3_In_2_ reveals notable differences in their deformation mechanisms and mechanical behaviors. Au_3_In demonstrates greater angular flexibility, primarily attributed to the dominance of bond bending, which allows it to adapt more effectively to angular shifts with minimal impact on bond length alterations. On the contrary, Au_3_In_2_ relies less on angular distortions, emphasizing adjustments in bond lengths during deformation. This characteristic results in a more balanced response between bond bending and bond stretching, leading to a mechanical response that is less flexible in terms of angular adaptability. These variations in deformation modes and the interplay of bond-related adjustments provide deeper insights into the structural and mechanical properties of these two crystals, showcasing the contrasting approaches they employ to accommodate external stresses and maintain structural integrity.

#### 3.2.5. Mechanical Properties

The bulk modulus (K) is a measure of the resistance of a material to volumetric changes under applied pressure, whereas the shear modulus (G) represents the resistance to reversible deformations caused by shearing stresses [46]. Young’s modulus (E), on the other hand, is defined as the ratio of tensile stress to tensile strain and serves as a key indicator of elasticity. Specifically, it quantifies the resistance of an elastic material to any alteration in its length [47], thereby also providing a measure of thermal shock resistance. In general, for a given class of solids, higher values of E indicate greater stiffness, signifying a more rigid material with superior mechanical performance. Poisson’s ratio (v), which ranges from −0.5 to 0.5, is a measure of the shape change of a material under an applied load, essentially capturing the ratio between transverse strain and axial strain experienced by a material under axial loading conditions. An increase in v suggests enhanced plasticity, which is highly relevant in engineering contexts where deformability is crucial. These polycrystalline mechanical properties are of paramount importance for engineering applications and can be described effectively as functions of the independent elastic coefficients through the Voigt–Reuss method [48]:

(14) $$K_{V}=\frac{1}{9}\left[C_{11}+C_{22}+C_{33}+2(C_{12}+C_{13}+C_{23})\right],$$

(15) $$K_{R}={\left[S_{11}+S_{22}+S_{33}+2(S_{12}+S_{13}+S_{23})\right]}^{-1},$$

(16) $$G_{V}=\frac{1}{15}\left[C_{11}+C_{22}+C_{33}-(C_{12}+C_{13}+C_{23})+3(C_{44}+C_{55}+C_{66})\right],$$

(17) $$G_{R}=15{\left[4\left(S_{11}+S_{22}+S_{33})-4(S_{12}+S_{13}+S_{23}\right)+3(S_{44}+S_{55}+S_{66})\right]}^{-1},$$

where *K_V_* and *K_R_* stand for the upper (Voigt) and lower (Reuss) bounds of the *K* of the polycrystalline aggregate, respectively, and *G_V_* and *G_R_* represent those of the *G*. Furthermore, the effective *K* and *G* can be assessed by using the Voigt–Reuss–Hill approximation [26], which is referred as a geometric mean:

(18) $$K=\sqrt{K_{V}\cdot K_{R}},$$

(19) $$G=\sqrt{G_{V}\cdot G_{R}},$$

Additionally, through the calculated polycrystalline *K* and *G*, the effective *E* and *ν* of the Au_3_In and Au_3_In_2_ IMCs can be calculated as follows:

(20) $$E=\frac{9KG}{3K+G},$$

(21) $$v=\frac{3K-2G}{2(3K+G)},$$

The calculated *K*, *G*, *E*, and *v* for polycrystalline Au_3_In and Au_3_In_2_ are comprehensively presented in Table 5. The obtained results clearly indicate that all of the mechanical properties, including *K*, *G*, and *E*, for Au_3_In substantially exceed those of Au_3_In_2_, thereby suggesting a significantly superior resistance to deformation under hydrostatic, shear, and tensile loading conditions for Au_3_In_2_. These results emphasize the superior mechanical robustness and ductility of Au_3_In compared to Au_3_In_2_, underscoring its ability to better accommodate stress without fracturing. Such mechanical advantages are particularly critical in microelectronic packaging, where materials are routinely subjected to thermal cycling, mechanical shock, and vibration. The improved deformation tolerance of Au_3_In can help mitigate crack initiation and propagation, thereby enhancing the long-term reliability and durability of the solder joints. Consequently, the mechanical behavior of Au_3_In makes it a more favorable candidate for high-reliability packaging applications than Au_3_In_2_.

To differentiate the physical properties of materials, Pugh [49] introduced an empirical relationship based on the ratio of *K* to *G*, termed the *K/G* ratio, to characterize ductile and brittle behaviors. The critical value for this ratio is approximately 1.75, above which materials are generally classified as ductile, whereas those with a ratio below this threshold typically exhibit brittle characteristics. The calculated *K/G* ratios for polycrystalline Au_3_In and Au_3_In_2_ are 5.285 and 4.489, respectively, both of which significantly and clearly exceed the threshold value of 1.75, thereby indicating that Au_3_In and Au_3_In_2_ very clearly exhibit evident ductile behavior. This particular insight is quite crucial for properly assessing their overall suitability in various different applications where mechanical properties are of utmost importance and significance. It is also important to note that the empirical relationship introduced by Pugh is highly regarded and frequently utilized in the field of material science to make determinations about the nature of different materials.

In the field of abrasive wear-resistant applications, the hardness of materials assumes a critically important and fundamental role. Teter et al. [50], in his influential work, proposed a linear relationship between Vickers hardness, represented as H_V_, and the shear modulus, particularly in cases where the material exhibits intrinsic brittleness. This linear correlation suggests that an increase in the shear modulus of brittle materials corresponds to a direct increase in their Vickers hardness. It is essential to emphasize that material hardness is not exclusively dependent on the shear modulus. Hardness is also inherently connected to the bulk modulus in a wide range of materials spanning various classes. This dual dependency on both shear modulus and bulk modulus has been a subject of significant research and analysis.

Chen et al. [51], building upon previous investigations, introduced a more sophisticated and enhanced non-linear correlation model, incorporating both bulk modulus and shear modulus to provide a more accurate and comprehensive representation of the relationship between elastic moduli and hardness. However, despite the improvements introduced in this model, it was subsequently identified that, under specific circumstances, this non-linear correlation could result in an unrealistic outcome, particularly yielding a negative Vickers hardness value. This issue arose primarily due to the inclusion of the final correlation term, expressed as “−3”. Such results raised concerns about the model’s applicability across all types of materials, prompting further revisions.

To address and mitigate this issue, Tian et al. [52] later introduced a modified correlation model, which was specifically designed to alleviate and reduce the likelihood of such problematic outcomes. The revised model is represented by the following equation:

(22) $$H_{v}=0.92\alpha^{1.137}G^{0.708},\;\alpha =G/K,$$

This formula provides a more accurate and reliable prediction of Vickers hardness across a wider range of materials. Tian’s modification not only resolves the concern about negative hardness values but also ensures that the model remains valid for a broader spectrum of materials, thus improving its applicability in practical scenarios where hardness estimation is crucial for design and material selection. Materials with a Vickers hardness exceeding 40 GPa are typically classified as superhard materials [53]. The calculated Vickers hardness values for the polycrystalline Au_3_In and Au_3_In_2_ IMCs, as presented in Table 7, are significantly lower than the threshold required for classification as superhard materials. This observation indicates that both Au_3_In and Au_3_In_2_ possess relatively low densification characteristics, which implies that these materials may not be suitable for applications requiring high hardness and wear resistance. Furthermore, the results reveal that the Vickers hardness of Au_3_In_2_ is slightly greater than that of Au_3_In, suggesting that Au_3_In_2_ inherently exhibits higher hardness compared to Au_3_In. This subtle difference in hardness may be attributed to variations in atomic bonding strength and crystal structure between the two IMCs.

The hardness of Au_3_In_2_ was further compared with the values reported in the literature [17] to assess the reliability of our results. Specifically, the literature reports a hardness of Au_3_In_2_ as 1.83 GPa, whereas our calculations yielded a value of 1.54 GPa. The close proximity of these values indicates that our computational model is capable of accurately predicting the mechanical properties of Au_3_In_2_. The slight discrepancy, where the hardness reported in the literature is marginally higher, may be attributed to differences in sample conditions. Notably, the value in the literature pertains to Au_3_In_2_ in a hardened state, which inherently leads to an increase in hardness. Such treatment typically involves processes that induce strengthening mechanisms, thereby elevating the hardness beyond that of the untreated or differently prepared phases. The consistency of our results with the literature demonstrates the robustness of our modeling approach and suggests that the discrepancies are primarily due to the physical condition of the material rather than a fundamental issue with our methodology.

Niu et al. [54] developed a precise and comprehensive fracture toughness model applicable to covalent and ionic crystals, metals, as well as IMCs. This model accounts for the fundamental differences between these material types by introducing enhancement factors, denoted as α. These factors are composed of two key components: the relative density of states at the Fermi level and the atomic electronegativity of the material. The incorporation of these parameters enables the model to capture the distinctive bonding characteristics and electronic structures of different materials. In particular, the model for IMCs is formulated to address their unique bonding nature, which involves contributions from both metallic and non-metallic elements. The calculation framework for determining the fracture toughness of IMCs is presented as follows:

(23) $$K_{IC}=(1+\alpha )\cdot V^{1/6}\cdot G\cdot {\left(K/G\right)}^{1/2},$$

(24) $$\alpha =43g{\left(E_{F}\right)}_{R}^{1/4}\cdot f_{EN},$$

(25) $$g{\left(E_{F}\right)}_{R}=g(E_{F})/g{\left(E_{F}\right)}_{FES},\;g{\left(E_{F}\right)}_{FES}=0.025,$$

(26) $$f_{EN}=\beta /{\left[1+\frac{C_{m}^{1}C_{n}^{1}}{C_{m+n}^{2}}\cdot \sqrt{\frac{{\left(x_{A}-x_{B}\right)}^{2}}{x_{A}\cdot x_{B}}}\right]}^{\gamma },$$

*V* represents the volume of each unit cell, while *K* and *G* denote the modulus shear and bulk modulus, respectively. The enhancement factor α is employed to differentiate between covalent crystals, ionic crystals, and metals, accounting for the distinct bonding characteristics in these materials. The term *g(E_F_)_R_* refers to the relative density of states of the alloy, with *g(E_F_)* representing the density of states at the Fermi level for the alloy and *g(E_F_)_FES_* corresponding to the density of states for the hydrogen atom. Additionally, the factor *f_EN_* addresses the role of electronegativity in influencing the properties of the material. For instance, in the case of Cu and P, the electronegativity values are *x_A_* = 2.54 for Au and *x_B_* = 1.78 for In. These values reflect the tendency of atoms to attract electrons, influencing the bonding strength. The parameters *β* and *γ*, which are determined through data fitting for IMCs, are found to be 0.3 and 8, respectively. For a compound *A_m_B_n_*, $C_{m}^{1}$, $C_{n}^{1}$, and $C_{m+n}^{2}$ refer to the number of possible combinations, providing a framework for calculating the interactions between the constituent elements.

Table 7 shows the calculated fracture toughness values for the Au_3_In and Au_3_In_2_ IMCs, which are 0.999 and 0.815, respectively. These values indicate that Au_3_In has a noticeably higher fracture toughness compared to Au_3_In_2_. The difference in fracture toughness suggests that the atomic bonding strength within the crystal structures of these two compounds plays a significant role in determining their mechanical performance. Au_3_In, with a higher fracture toughness value, is likely to be more resistant to crack initiation and propagation under mechanical loading, providing greater reliability in applications that require enhanced durability. On the other hand, Au_3_In_2_, with a lower fracture toughness, may be more susceptible to fracture under similar conditions. Moreover, the two values for Au_3_In and Au_3_In_2_ IMCs are consistent with the typically low toughness associated with IMCs, as reported by Niu et al. [54]. This characteristic brittleness of IMCs is often attributed to their complex crystal structures and limited capacity for plastic deformation, which makes them prone to crack propagation under stress. Consequently, while both Au_3_In and Au_3_In_2_ exhibit low fracture toughness values, understanding these limitations is vital for optimizing solder joint reliability, especially in electronic packaging applications where the mechanical stability of IMCs plays a critical role in the overall performance.

The index *δ* serves as a valuable tool for assessing the plasticity of a material [55], as well as evaluating its dry lubricating properties. The formulation of this index is given by

(27) $$\delta =\frac{K}{C_{11}},$$

This formulation indicates that a combination of high bulk strength and low shear resistance is advantageous for achieving enhanced dry lubricity. Specifically, a material characterized by a large value of *δ* is indicative of superior dry lubricating properties, reduced frictional forces, and an elevated plastic strain capacity. Such attributes are particularly important in determining the effectiveness of the material under dry conditions, where minimizing friction and maximizing plastic deformation are essential to ensuring overall performance and durability. Consequently, the δ ratio provides crucial insights into both the deformation behavior and lubricating efficiency of the material, contributing to a more comprehensive understanding of its mechanical properties. The δ values for Au_3_In and Au_3_In_2_, as presented in Table 7, are 4.92 and 3.76, respectively. From this comparison, it becomes even more evident that the δ value of Au_3_In is substantially greater than that of Au_3_In_2_, clearly indicating that Au_3_In exhibits significantly more obvious plastic characteristics. This important observation closely aligns with the results obtained from both the Cauchy pressure and K/G ratio analyses, which consistently confirm and further validate that Au_3_In demonstrates superior ductility.

### 3.3. Characterization of Elastic Anisotropic Properties

#### 3.3.1. Anisotropy Indexes

The elastic anisotropy observed in crystalline materials exerts a profound influence on a wide range of physical properties, including anisotropic plastic deformation, crack propagation behavior, and elastic instability. Such anisotropy can affect how a material responds to mechanical stresses, influencing not only its durability but also its performance under various loading conditions. The inherent elastic anisotropy of a crystal is largely dictated by the differences in bonding strength and atomic arrangement across different crystallographic planes and directions. This characteristic is particularly important in understanding the mechanical response of materials in engineering applications, where directional properties can play a critical role in determining the structural integrity of components. One of the key ways to quantify and evaluate the extent of elastic anisotropy is through the calculation of shear anisotropy factors. These factors provide a valuable measure of the degree of anisotropy in atomic bonding strength across various planes of the crystal. Specifically, the shear anisotropy factor serves as a metric for assessing how the bonding strength varies when shear forces are applied along different crystallographic planes and directions. The magnitude of these factors can offer insights into the directional dependence of mechanical properties, such as how a material might deform plastically or fracture under certain loading scenarios.

In the study, the universal anisotropy index (*A^U^*), equivalent Zener anisotropy index (*A^eq^*), the universal log-Euclidean anisotropy index (*A^L^*), shear anisotropic indexes, and percentage of anisotropy in compressibility and shear are investigated. The universal anisotropy index, which provides a measure of anisotropy independent of the crystal symmetry, can be determined using the following equation [56]:

(28) $$A^{U}=5\frac{G_{V}}{G_{R}}+\frac{K_{V}}{K_{R}}-6,$$

*G*_V_, *G*_R_, *K*_V_ and *K*_R_ are calculated by Voigt bounds and Reuss bounds, respectively. According to Equation (27), a larger fractional difference between the Voigt and Reuss estimates for bulk or shear modulus signifies a greater degree of anisotropy within the crystal structure. Based on the values of *G*_V_/G_R_ and *K*_V_/*K*_R_, it is evident that *G*_V_/*G*_R_ exerts a more significant influence on the universal anisotropy index compared to *K*_V_/*K*_R_. The universal anisotropy index can only assume values that are zero or positive. A value of zero indicates that the crystal is isotropic, while any deviation from zero reflects the presence of anisotropy. The calculated results presented in Table 7 reveal that the universal anisotropy index for both Au_3_In and Au_3_In_2_ is non-zero, thereby confirming the anisotropic behavior of these two intermetallic compounds. Specifically, the *A^U^* value for Au_3_In is determined to be 0.24, whereas that for Au_3_In_2_ is 2.83. The greater deviation of the *A^U^* value of Au_3_In_2_ from zero indicates a significantly higher degree of anisotropy in comparison to Au_3_In. This observation implies that Au_3_In_2_ exhibits a more distinct anisotropic character in its mechanical properties than Au_3_In.

The equivalent Zener anisotropy index can be expressed by the following equation [57]:

(29) $$A^{eq}=(1+\frac{5}{12}A^{U})+\sqrt{{\left(1+\frac{5}{12}A^{U}\right)}^{2}-1},$$

It can be observed that the equivalent Zener anisotropy index is calculated based on the value of the universal anisotropy index. For an isotropic crystalline material, the equivalent Zener anisotropy index is exactly equal to one. However, the *A^eq^* values for both Au_3_In and Au_3_In_2_, as presented in Table 7, deviate from one, indicating that both IMCs inherently exhibit anisotropic behavior. Moreover, the *A^eq^* value for Au_3_In_2_ is farther from one compared to that of Au_3_In, suggesting that Au_3_In_2_ exhibits a significantly higher degree of anisotropy. This disparity further emphasizes the distinct mechanical behavior of Au_3_In and Au_3_In_2_, reinforcing the conclusion that Au_3_In_2_ is more anisotropic than Au_3_In in terms of its structural and elastic properties.

The universal log-Euclidean anisotropy index, applicable to all crystal symmetries, can be expressed as follows [58]:

(30) $$A^{L}=\sqrt{{\left[\ln\left(\frac{K_{V}}{K_{R}}\right)\right]}^{2}+5{\left[\ln\left(\frac{G_{V}}{G_{R}}\right)\right]}^{2}},$$

A crystal is regarded as perfectly isotropic when the log-Euclidean anisotropy index is equal to zero. The calculated *A^L^* values for Au_3_In and Au_3_In_2_ are 0.10 and 1.00, respectively, as displayed in Table 7. It is evident from these values that neither Au_3_In nor Au_3_In_2_ possesses an *A^L^* value of zero, which clearly indicates that both materials exhibit anisotropic behavior. Moreover, the *A^L^* value for Au_3_In_2_ deviates more significantly from zero compared to Au_3_In, implying that Au_3_In_2_ has a notably higher degree of anisotropy than Au_3_In. Generally, *A^L^* values range from 0 to 10.26, with nearly 90% of solids exhibiting *A^L^* values greater than one, indicating varying levels of anisotropy among materials. Additionally, it has been argued that the *A^L^* index can also serve as an indicator of the presence of a layered or lamellar structure within a crystalline material [58]. Specifically, materials characterized by evident layered structures are typically associated with higher *A^L^* values, whereas those without such features exhibit comparatively lower *A^L^* values. From the calculated *A^L^* values for Au_3_In and Au_3_In_2_, it can be inferred that both IMCs do not demonstrate a significant layered structural configuration, as their *A^L^* values are relatively low.

The percentage of elastic anisotropy in both compressibility and shear can be quantified using the following two dimensionless parameters [57]:

(31) $$A^{K}=\frac{K_{V}-K_{R}}{K_{V}+K_{R}},$$

and

(32) $$A^{G}=\frac{G_{V}-G_{R}}{G_{V}+G_{R}}.$$

A zero value for *A*^K^ and *A*^G^ indicates isotropic behavior, while a value of 100% represents the theoretical upper limit for anisotropy. The calculated results for both Au_3_In and Au_3_In_2_ are presented in Table 7. From the results, it is evident that Au_3_In exhibits a greater degree of anisotropy in compressibility compared to Au_3_In_2_, indicating a significant variation in bonding strength under volumetric stress across different crystallographic directions. However, Au_3_In demonstrates a lower degree of anisotropy in shear response compared to Au_3_In_2_, implying a more uniform resistance to shear deformation across various planes. This clear contrast reveals the complex anisotropic mechanical behavior inherent in both IMCs, which is influenced by the underlying crystal structure and bonding characteristics, thereby affecting their potential suitability for advanced engineering applications.

To provide a more detailed elucidation of the characteristics of shear anisotropy, the shear anisotropic factor is introduced, as it offers a quantitative metric to assess the degree of anisotropy in atomic bonding along various crystallographic directions in crystal planes. This factor serves as a precise indicator of the anisotropy present in the bonding between atoms in different crystal planes [59]. Specifically, the shear anisotropic factor for the {100} shear plane between the <011> and <010> crystallographic directions is

(33) $$A_{1}=\frac{4C_{44}}{C_{11}+C_{33}-2C_{13}},$$

for the {010} shear planes between the <101> and <001> directions,

(34) $$A_{2}=\frac{4C_{55}}{C_{22}+C_{33}-2C_{23}},$$

and for the {001} shear planes between the <110> and <010 > directions,

(35) $$A_{3}=\frac{4C_{66}}{C_{11}+C_{22}-2C_{12}},$$

In the case of isotropic crystals, *A*_1_, *A*_2_, and *A*_3_, are all equal to unity, while any deviation from unity represents the amplitude of anisotropy of the crystal. The calculated shear anisotropy factors for Au_3_In and Au_3_In_2_ are presented in Table 7, revealing that the values for the {010} shear plane of Au_3_In and the {001} shear plane of Au_3_In_2_ are both approximately equal to 1. This suggests that these specific shear planes in Au_3_In and Au_3_In_2_ exhibit isotropic behavior. In contrast, the remaining shear planes display notable elastic anisotropy, indicating variations in their mechanical response depending on the crystallographic direction. This analysis provides deeper insights into the anisotropic mechanical properties of Au_3_In and Au_3_In_2_, particularly in terms of their shear behavior across different crystallographic planes.

#### 3.3.2. Directional Mechanical Properties

Crystal orientation plays a crucial role in influencing crystal anisotropy, making it essential to carefully consider the elastic anisotropy in each specific direction. In this study, the changes in three-dimensional (3D) surface construction are used as an intuitive means of illustrating the directional dependence of elastic anisotropy. The calculated directional elastic anisotropy provides a clear visualization of how mechanical properties vary with respect to crystallographic orientation, offering a comprehensive understanding of the anisotropic behavior of the material. The directional relationship of the Young’s modulus, shear modulus, and Poisson’s ratio were explored using the following equations [60]:

(36) $$E(\theta ,\varphi )=\frac{1}{{S^{\prime }}_{11}(\theta ,\varphi )}=\frac{1}{a_{i}a_{j}a_{k}a_{l}S_{ijkl}},$$

(37) $$G(\theta ,\varphi ,\chi )=\frac{1}{4{S^{\prime }}_{66}(\theta ,\varphi ,\chi )}=\frac{1}{4a_{i}b_{j}a_{k}b_{l}S_{ijkl}},$$

and

(38) $$v(\theta ,\varphi ,\chi )=-\frac{{S^{\prime }}_{12}(\theta ,\varphi ,\chi )}{{S^{\prime }}_{11}(\theta ,\varphi )}=-\frac{a_{i}a_{j}b_{k}b_{l}S_{ijkl}}{a_{i}a_{j}a_{k}a_{l}S_{ijkl}},$$

where *S_ijkl_* stands for the compliance coefficients, and *a* and *b* are the unit vectors. The unit vector a needs two angles, *θ* and *φ*, to describe it. In addition, the shear modulus requires another unit vector, *b*, which is characterized by the angle *χ*. Moreover, unit vector *b* is perpendicular to unit vector *a*. The *θ* is in the range of 0~π, and *ϕ* and *χ* are in the range of 0~2π. The coordinates of two vectors, a and b, are shown blow,

(39) $$a=\left(\begin{array}{c} \sin\theta \cos\varphi \\ \sin\theta \sin\varphi \\ \cos\theta \end{array}\right),\;b=\left(\begin{array}{c} \cos\theta \cos\varphi \cos\chi -\sin\theta \sin\chi \\ \cos\theta \sin\varphi \cos\chi +\cos\theta \sin\chi \\ -\sin\theta \cos\chi \end{array}\right).$$

**Figure 4:**
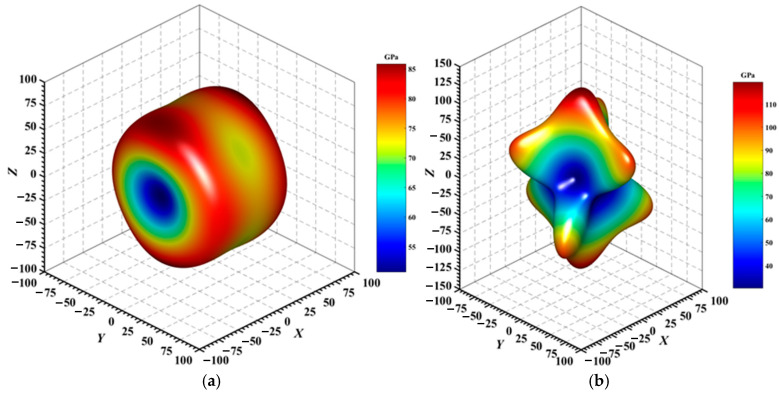
3D surface representation of the Young’s modulus of (**a**) Au_3_In and (**b**) Au_3_In_2_.

**Figure 5:**
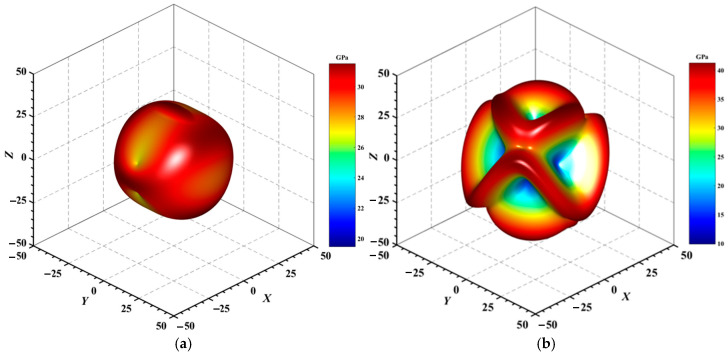
3D surface representation of the maximum shear modulus of (**a**) Au_3_In and (**b**) Au_3_In_2_.

**Figure 6:**
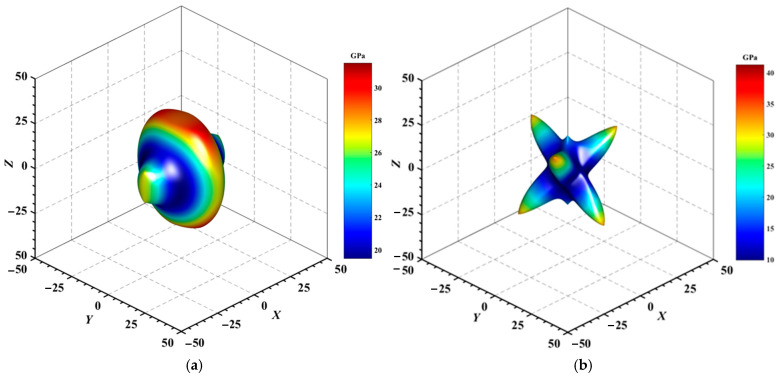
3D surface representation of the minimum shear modulus of (**a**) Au_3_In and (**b**) Au_3_In_2_.

**Figure 7:**
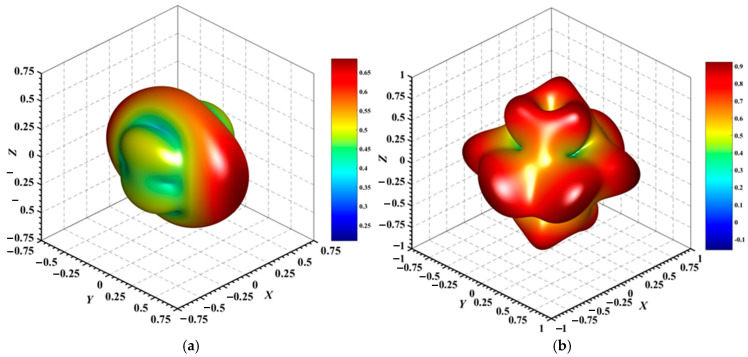
3D surface representation of the maximum Poisson’s ratio of (**a**) Au_3_In and (**b**) Au_3_In_2_.

**Figure 8:**
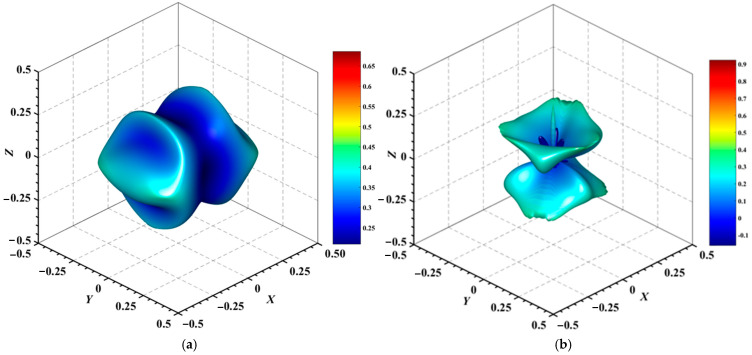
3D surface representation of the minimum Poisson’s ratio of (**a**) Au_3_In and (**b**) Au_3_In_2_.

**Figure 9:**
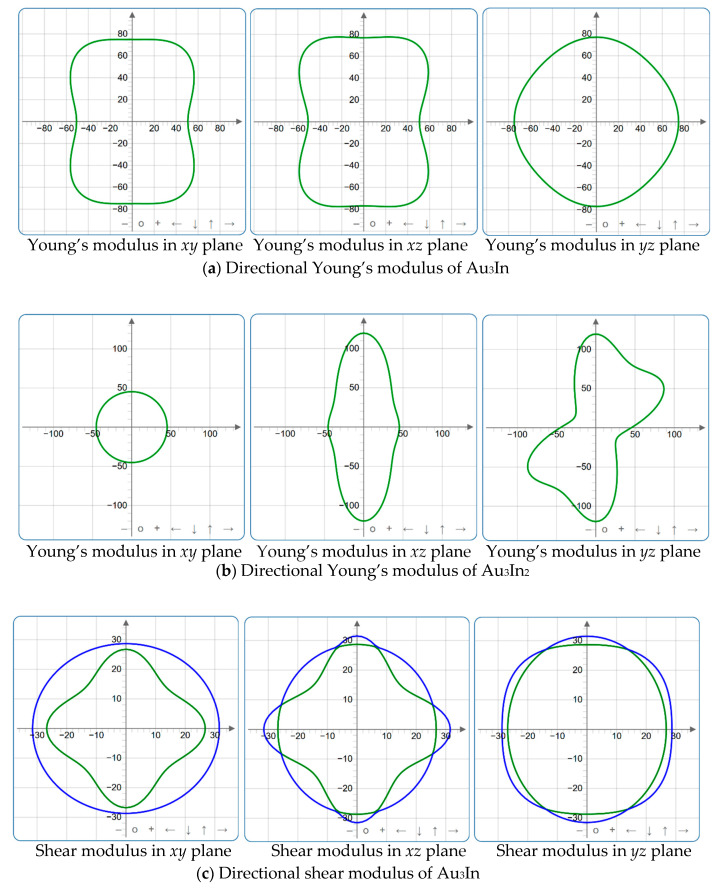

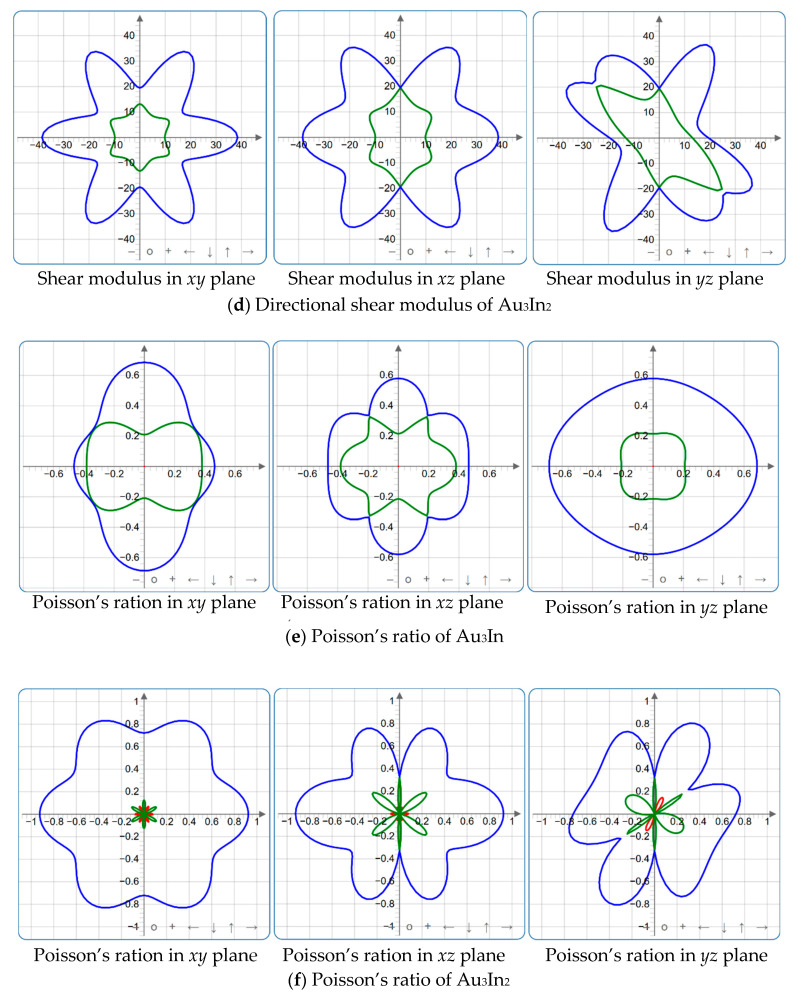
2D projections in *xy*, *xz* and *yz* planes of Young’s modulus, shear modulus, and Poisson’s ratio of Au_3_In and Au_3_In_2_.

Figure 4, Figure 5, Figure 6, Figure 7, Figure 8 and Figure 9 provide an elaborate graphical representation of the vectors, their angular relationships, and their two-dimensional projections on the *xy*, *xz*, and *yz* planes. These illustrations are essential for elucidating the directional characteristics of Au_3_In and Au_3_In_2_ crystals, facilitating a comprehensive understanding of their anisotropic properties, which markedly differ from the isotropic nature of a perfect sphere. The directional variations observed in Young’s modulus, shear modulus, and Poisson’s ratio indicates a consistently higher degree of anisotropy in Au_3_In_2_ compared to Au_3_In. Specifically, for the shear modulus and Poisson’s ratio, the blue curve represents the maximal values, while the green curve depicts the minimal values. Figure 4 and Figure 9b further provide additional insights into the directional variation of Young’s modulus for Au_3_In_2_, showcasing notable patterns across different crystallographic planes. In comparison, Au_3_In demonstrates relatively uniform behavior, indicating a lower level of anisotropy. Similarly, the two-dimensional projections of the shear modulus, shown in Figure 9c,d, reveal contrasting characteristics between Au_3_In and Au_3_In_2_. For Au_3_In_2_, significant variations are observed across various crystallographic planes, reflecting its evident anisotropic behavior. In contrast, Au_3_In exhibits relatively uniform values across the same planes, suggesting a more isotropic nature in its shear modulus. This comparison demonstrates the notable difference in anisotropic properties between the two materials. Regarding Poisson’s ratio, Figure 9e,f indicate that Au_3_In_2_ exhibits significantly greater anisotropy compared to Au_3_In.

To gain a more comprehensive understanding of the elastic anisotropy of Au_3_In and Au_3_In_2_, the maximum and minimum values of *E*_max_, *E*_min_, *G*_max_, *G*_min_, *v*_max_, and *v*_min_, along with the corresponding ratios *E*_max_/*E*_min_, *G*_max_/*G*_min_, and *v*_max_/*v*_min_, have been systematically analyzed. The results are summarized in Table 8. For isotropic materials, the ratios of the maximum to minimum elastic moduli are equal to unity. In contrast, for anisotropic materials, these ratios deviate from unity, with larger values indicating a higher degree of anisotropy. The results indicate that the ratios *E*_max_/*E*_min_, *G*_max_/*G*_min_, and *v*_max_/*v*_min_ for Au_3_In_2_ are 3.98, 4.18, and 5.75, respectively, all of which are significantly greater compared to the corresponding values for Au_3_In. This further demonstrates the considerably higher degree of anisotropy exhibited by Au_3_In_2_ in comparison to Au_3_In.

### 3.4. Thermophysical Properties

#### 3.4.1. Debye Temperature

The Debye temperature (*Θ_D_*) serves as a pivotal parameter that influences an extensive range of thermal properties in solids [61,62,63]. Numerous thermophysical properties, such as thermal conductivity, lattice vibrations, interatomic bonding strength, melting temperature, coefficient of thermal expansion, phonon-specific heat capacity, and elastic constants, are intricately tied to the Debye temperature. Typically, materials with stronger interatomic bonding, higher melting points, greater hardness, elevated mechanical wave velocity, and lower average atomic mass tend to exhibit higher Debye temperatures. When the temperature surpasses Debye temperature, all vibrational modes possess an energy approximately equivalent to *k_B_T*, whereas at temperatures below Debye temperature, the higher frequency modes become frozen, giving rise to the manifestation of the quantum nature of vibrational modes [64]. Interestingly, the Debye temperature calculated using elastic constants aligns closely with that determined from specific heat measurements at low temperatures. This consistency can be attributed to the fact that, at low temperatures, vibrational excitations are largely governed by acoustic modes. Such observations emphasize the profound significance of Debye temperature in capturing the thermal essence of materials, offering deeper insights into their intricate response to fluctuations in temperature and revealing the quantum underpinnings of their behavior. The Debye temperature of a material, which is directly proportional to the average sound velocity, can be derived from the following relationship [65]:

(40) $$\Theta_{D}=\frac{\hbar }{k_{B}}{\left[\frac{3n}{4\pi }\left(\frac{N_{A}\rho }{M}\right)\right]}^{\frac{1}{3}}V_{m},$$

ℏ is Planck’s constant, *k_B_* is the Boltzmann constant, n expresses the atomic numbers within the unit cell, *N_A_* is the Avogadro constant, ρ is the density, and the molecular weight is represented by M. The transverse sound velocity *V_t_* and longitudinal sound velocity *V_l_* can calculate the average sound velocity *V_m_*, and the expressions are as follows [66]:

(41) $$V_{m}={\left[\frac{1}{3}\left(\frac{2}{V_{t}^{3}}+\frac{1}{V_{l}^{3}}\right)\right]}^{-\frac{1}{3}},$$

(42) $$V_{l}={\left[\frac{\left(K+\frac{4G}{3}\right)}{\rho }\right]}^{\frac{1}{2}},$$

(43) $$V_{t}={\left(\frac{G}{\rho }\right)}^{\frac{1}{2}}.$$

As indicated in Table 9, the Debye temperature of Au_3_In is slightly higher, reaching 165.3 K, compared to Au_3_In_2_, which has a lower Debye temperature of 157.9 K. Moreover, there is a positive correlation between Debye temperature and thermal conductivity, meaning that materials with higher Debye temperature generally exhibit superior thermal conductivity. Consequently, Au_3_In shows greater thermal conductivity than Au_3_In_2_, reflecting the stronger bonding and more effective heat conduction properties of Au_3_In.

#### 3.4.2. Coefficients of Thermal Expansion

Thermal-induced stress in electronic packaging originates from differences in the coefficients of thermal expansion (CTE) between the materials comprising the package structure. When subjected to thermal cycling, these mismatches give rise to thermal stress, which, over extended periods, may lead to the development of cracks at the solder joints and other structural components, thereby diminishing the overall reliability of the system. Specifically, at the solder and IMC interface, thermal-induced stress can be characterized by the following equation,

(44) $$\sigma =\frac{E(\alpha_{\mathrm{IMC}}-\alpha_{\mathrm{solder}})\Delta T}{1-v},$$

where *σ* is the thermal stress, *α_IMC_* is the CTE of the IMC, *α_solder_* is the CTE of the solder, Δ*T* is the difference in temperature, and *v* is Poisson’s ratio. The equation clearly demonstrates that the magnitude of thermal stress is intricately linked to the coefficient of thermal expansion. Consequently, to ensure the reliability of electronic packaging systems, a comprehensive understanding of the CTE of the materials involved is crucial. In light of this, this study undertakes an in-depth investigation into the thermal expansion coefficients of Au_3_In and Au_3_In_2_, aiming to provide valuable insights into their behavior under thermal stress and to enhance the overall reliability of packaging applications. The thermal expansion coefficient of a material can be calculated as follows [67]:

(45) $$\alpha =\frac{1.6\times 10^{-3}}{G}.$$

The results for the coefficient of thermal expansion are presented in Table 9. It can be observed from the table that the CTE of Au_3_In_2_ is higher than that of Au_3_In, indicating that Au_3_In_2_ endure a greater degree of expansion when subjected to thermal exposure. This suggests that Au_3_In_2_ exhibits a higher thermal expansion response compared to Au_3_In, which is indicative of the differing thermal behaviors of both IMCs.

#### 3.4.3. Melting Point

The melting temperature, also referred to as the melting point, is the specific temperature at which a material transitions from the solid to the liquid phase under a defined atmospheric pressure. In contemporary research, the melting temperature (*T_m_*) of a material represents a crucial and compelling area of study, as it plays a significant role in determining the material suitability for various high-temperature applications. The melting temperature can be further calculated by employing the formula given below [68]:

(46) $$T_{m}=354+1.5(2C_{11}+C_{33}).$$

The calculated values of *T_m_* for Au_3_In and Au_3_In_2_ are presented in Table 9. The results indicate that the melting points of both IMCs exceed 1000 K, reflecting their considerable thermal stability within the temperature range typically encountered during semiconductor packaging processes. Moreover, Au_3_In exhibits a slightly higher melting point compared to Au_3_In_2_, suggesting a marginal difference in their thermal robustness, potentially arising from subtle variations in atomic bonding characteristics and crystallographic structure. This minor difference in melting behavior may influence their applicability and reliability in advanced microelectronic packaging technologies, where thermal stability is critical.

#### 3.4.4. Heat Capacity

Heat capacity represents a fundamental thermodynamic property intrinsic to a material. Systems with elevated heat capacity generally show enhanced thermal conductivity and reduced thermal diffusivity. The volumetric heat capacity (*ρ*C_p_) is defined as the amount of thermal energy change per unit volume for a given temperature change, measured in degrees Kelvin. At elevated temperatures, the volumetric heat capacity can be calculated through the application of the following equation [64]. This parameter plays a critical role in determining how efficiently a material can store and transfer thermal energy, influencing its overall thermal performance in various applications.

(47) $${\rho C}_{p}=\frac{3k_{B}}{\Omega },$$

*Ω* is volume occupied by an atom. Table 9 presents the calculated results for the volumetric heat capacity. It can be observed that the volumetric heat capacity of Au_3_In is higher than that of Au_3_In_2_. This difference indicates that Au_3_In has a greater capacity to store thermal energy per unit volume compared to Au_3_In_2_, reflecting variations in their thermophysical properties. The higher volumetric heat capacity of Au_3_In suggests that it may show superior thermal performance under specific conditions, which is significant in applications where thermal management is critical.

#### 3.4.5. Dominant Phonon Wavelength

Phonons, which are quantized lattice vibrations in materials, significantly influence various physical properties such as electrical conductivity, thermoelectric power, thermal conductivity, and heat capacity. In metals and semiconductors, phonon–electron interactions play a critical role in electrical transport. At low temperatures, electron–phonon scattering is relatively weak, and impurity scattering dominates. However, as temperature increases, the phonon population rises, leading to more frequent electron–phonon collisions, which increase resistivity and reduce electrical conductivity [69]. In thermoelectric materials, phonon interactions affect charge carrier mobility, thereby influencing thermoelectric efficiency [70]. Additionally, phonons contribute to thermal conductivity via lattice vibrations, where their mean free path determines heat transport efficiency [71]. Heat capacity is also governed by phonon behavior, as described by the Debye model, where vibrational modes contribute to specific heat at different temperature regimes [72]. The dominant phonon wavelength, represented as λ_dom_, refers to the wavelength where the phonon distribution function reaches its maximum. For Au_3_In and Au_3_In_2_ at 300 K, the dominant phonon wavelength has been estimated using the following equation [67]:

(48) $$\lambda_{\mathrm{dom}}=\frac{12.566V_{m}}{T}\times 10^{-12},$$

*T* is the temperature in degree Kelvin. Materials characterized by a higher average sound velocity, greater shear modulus, and lower density generally exhibit longer dominant phonon wavelengths [67]. The values of the dominant phonon wavelength, calculated for Au_3_In and Au_3_In_2_, are presented in Table 9. The *λ_dom_* for Au_3_In is determined to be 61.4 × 10^−12^ m, whereas for Au_3_In_2_, it is found to be 56.4 × 10^−12^ m. This indicates that Au_3_In exhibits a greater phonon wavelength dominance than Au_3_In_2_, suggesting variations in their lattice dynamics and mechanical behavior.

#### 3.4.6. Thermal Conductivity

The lattice thermal conductivity (*k_ph_*) can be defined as the measurement of the ability of a material to conduct heat via phonons through its crystal structure. It demonstrates how heat is efficiently conducted through a material as a result of the phonon propagation. High thermal conductivity materials are desirable for efficient heat dissipation for applications such as in electronic devices, whereas low thermal conductivity materials are useful for thermal barriers. In this study, we have calculated the k_ph_ of Au_3_In and Au_3_In_2_ compounds at room temperature (300 K) by employing the following empirical formula proposed by Slack [73]:

(49) $$k_{ph}=A(\gamma )\frac{M_{av}\Theta_{D}^{3}\delta }{\gamma^{2}n^{2/3}T},$$

where *M_av_* stands for the average atomic mass, *δ* refers to the cubic root of the average atomic volume, n denotes the total number of atoms in the unit cell, *γ* is the Grüneisen parameter, and *T* stands for the absolute temperature. The following relations [73,74] can be employed to obtain the parameter *γ* and the factor *A* (*γ*):

(50) $$\gamma =\frac{3(1+v)}{2(2-3v)},$$

(51) $$A(\gamma )=\frac{5.720\times 10^{7}\times 0.849}{2\times \left(1-0.514/\gamma +0.228/\gamma^{2}\right)}.$$

The calculated lattice thermal conductivities, presented in Table 9, demonstrate a difference between these two IMCs. In specific, the lattice thermal conductivity of the Au_3_In is higher compared to that of the Au_3_In_2_. This observation suggests that the Au_3_In exhibits superior thermal conductivity, thereby indicating a greater capacity for efficient heat transfer within its crystal structure. Such a discrepancy in thermal properties can be attributed to the inherent differences in atomic bonding, crystal arrangement, and phonon scattering mechanisms present in the two compounds. The higher lattice thermal conductivity of Au_3_In, compared to Au_3_In_2_, suggests its potential utility in applications requiring effective thermal management, particularly in high-performance environments where optimized thermal dissipation is critical.

For compounds intended for high-temperature applications, it is essential to understand the behavior of the solid at temperatures exceeding the Debye temperature. At elevated temperatures, the thermal conductivity of a material approaches a limiting value known as the minimum thermal conductivity (*k_min_*). This phenomenon occurs due to the complete decoupling of phonon modes, where heat is transferred solely to neighboring atoms. A key characteristic of minimum thermal conductivity is its independence from the presence of defects, such as dislocations, atomic vacancies, and long-range strain fields associated with impurity inclusions and dislocations. This independence arises because such defects primarily influence phonon transport over length scales significantly larger than the interatomic spacing, making their impact negligible at the atomic level. Understanding this fundamental limit is critical in predicting the thermal behavior of materials at high temperatures and in conditions where phonon scattering is prominent. Materials with higher sound velocity and Debye temperature exhibit high minimum thermal conductivity. In this study, Clark’s model [75] and Cahill’s model [76] are used to investigate the minimum thermal conductivity of Au_3_In and Au_3_In_2_.

Clarke’s model:

(52) $$k_{\min}=0.87k_{B}{M_{a}}^{-\frac{2}{3}}E^{\frac{1}{2}}\rho^{\frac{1}{6}},\;M_{a}=[M/(m\cdot N_{a})],$$

where *M_a_*, *M*, and *m* represent the average atomic mass, molar mass, and total number of atoms of the unit cell, respectively.

Cahill’s model:

(53) $$k_{\min}=\frac{k_{B}}{2.48}n^{\frac{2}{3}}(V_{l}+2V_{t}),$$

where *n* is the number of atoms per unit volume. Table 9 presents the values of *k_min_* calculated using Clarke’s and Cahill’s models. It is evident from the data that the *k_min_* values obtained from Clarke’s model are slightly lower than those derived from Cahill’s model. This discrepancy can be attributed to the fact that Clarke’s model disregards contributions from photons and phonons. However, despite this difference, the order of thermal conductivity values for each phase remains consistent across both calculation methods. The higher *k_min_* value of Au_3_In compared to Au_3_In_2_, as determined by both Clarke’s model (0.38 W/mK vs. 0.35 W/mK) and Cahill’s model (0.47 W/mK vs. 0.43 W/mK), demonstrates its enhanced thermal conductivity. This characteristic is particularly beneficial in high-temperature environments, as it facilitates the rapid dissipation of localized thermal stress. By effectively reducing stress concentrations during thermal cycles, Au_3_In is better equipped to mitigate the formation of thermal microcracks, thereby improving its reliability and performance under extreme conditions.

Moreover, thermal conductivity is strongly correlated with Debye temperature, as lattice thermal conductivity is intricately linked to the thermal behavior of the crystal [77]. In general, the total thermal conductivity is influenced by both the lattice and electronic thermal conductivity. At lower temperatures, electron–phonon scattering is minimized, resulting in lattice thermal conductivity becoming the dominant factor, though its value remains relatively low. According to the Callaway–Debye theoretical model [78], lattice thermal conductivity in low-temperature conditions is directly proportional to the Debye temperature. Consequently, materials with higher Debye temperature exhibit greater lattice thermal conductivity. This relationship is evident in the comparison between Au_3_In, which has the higher Debye temperature (165.3 K) and correspondingly higher thermal conductivity, and Au_3_In_2_, which has a lower Debye temperature (157.9 K) and, consequently, lower thermal conductivity.

The results derived from Slack’s model offer valuable insights into the phonon transport properties of Au_3_In and Au_3_In_2_ at moderate temperatures, where the contributions of intrinsic lattice vibrations dominate thermal conduction. In contrast, the thermal conductivity values obtained from Clarke’s and Cahill’s models emphasize the fundamental lower limits imposed by strong phonon scattering mechanisms, particularly Umklapp processes, which become increasingly significant at elevated temperatures. The interplay between these models provides a comprehensive framework for evaluating the thermal transport characteristics of these intermetallic compounds across a broad temperature range.

## 4. Conclusions

This study presents a comprehensive, first-principles DFT investigation into the mechanical and thermophysical properties of Au_3_In and Au_3_In_2_ intermetallic compounds, which play crucial roles in advanced microelectronic packaging technologies. The analyses revealed significant differences between these two IMCs in terms of mechanical stability, elastic anisotropy, and thermal transport properties, highlighting the superior structural resilience and thermal conductivity exhibited by Au_3_In.

Computed elastic moduli confirmed the mechanical stability of both Au_3_In and Au_3_In_2_. However, Au_3_In demonstrated a notably more robust structural framework characterized by a higher bulk modulus and reduced mechanical anisotropy. Furthermore, the evaluated Cauchy pressures indicated a higher degree of metallic bonding in Au_3_In, correlating well with its enhanced ductility compared to Au_3_In_2_. Additionally, the analysis based on the *K*/*G* ratio unequivocally classified both compounds as ductile, with Au3In possessing markedly greater plasticity, which is essential for mitigating mechanical failures under operational stress.

Elastic anisotropy assessments further indicated that Au_3_In_2_ exhibits significant directional dependence in its mechanical response, as evidenced by its elevated universal anisotropy index. This pronounced anisotropy suggests that applications utilizing Au_3_In_2_ would require precise crystallographic orientation control, particularly when mechanical performance is directionally sensitive. Conversely, the comparatively isotropic mechanical behavior of Au_3_In makes it well suited for scenarios requiring uniform performance under complex, multi-axial loading conditions.

From a thermophysical standpoint, the thermal transport properties of Au_3_In and Au_3_In_2_ are predominantly influenced by phonon interactions, especially Umklapp scattering processes that significantly resist heat conduction at elevated temperatures. The calculated lattice thermal conductivities at 300 K demonstrated that Au_3_In possesses a superior heat dissipation capability relative to Au_3_In_2_, attributed to its higher Debye temperature and stronger interatomic bonding, which enhance phonon group velocities and mitigate Umklapp scattering effects. Minimum thermal conductivity predictions via Clarke’s and Cahill’s models further reinforced the fundamental limitations imposed by Umklapp processes on phonon-mediated thermal transport in both compounds.

These results provide valuable insights into the intricate structure–property relationships governing Au_3_In and Au_3_In_2_ intermetallics, establishing a theoretical basis for their targeted application in microelectronic packaging technologies. Au_3_In emerges as the superior candidate for demanding applications requiring exceptional structural integrity and efficient thermal management, while the pronounced anisotropy of Au_3_In_2_ indicates its suitability for specialized applications emphasizing directional mechanical performance.

Future work could extend this foundational investigation by incorporating experimental validation of the predicted properties to establish correlations between theoretical predictions and practical performance. Further studies focusing on alloying and microstructural modifications could explore the potential for tuning mechanical and thermal properties to meet specific application requirements. Additionally, evaluating the long-term stability and reliability of these IMCs under realistic operational conditions, including thermal cycling and mechanical loading, will be crucial for advancing their practical application in next-generation microelectronic packaging solutions.

## Figures and Tables

**Table 1:** Reduced atomic coordinates for Au_3_In before relaxation.

Atom	*x*	*y*	*z*
Au	0.500	0.250	0.170
Au	0.250	0.750	0.330
In	0.250	0.250	0.667

**Table 2:** Reduced atomic coordinates for Au_3_In_2_ before relaxation.

Atom	*x*	*y*	*z*
Au	0.000	0.000	0.000
Au	0.333	0.667	0.154
In	0.333	0.667	0.640

**Table 3:** Reduced atomic coordinates for Au_3_In after relaxation.

Atom	*x*	*y*	*z*
Au	0.501	0.250	0.170
Au	0.250	0.750	0.328
In	0.250	0.250	0.671

**Table 4:** Reduced atomic coordinates for Au_3_In_2_ after relaxation.

Atom	*x*	*y*	*z*
Au	0.000	0.000	0.000
Au	0.333	0.667	0.194
In	0.333	0.667	0.680

**Table 5:** Calculated lattice constants *a*, *b,* and *c* (Å), equilibrium volume *V* (Å^3^), bulk modulus *K* (GPa), shear modulus *G* (GPa), Young’s modulus *E* (GPa), Poisson’s ratio *ν*, ratio of bulk modulus to shear modulus *K*/*G*, cohesive energy *E_c_* (eV/atom), and formation enthalpy Δ*H* (KJ/mol) of Au_3_In and Au_3_In_2_.

	Lattice Constants	*V*	*ζ*	*K*	*G*	*E*	*v*	*K/G*	*E_c_*	Δ*H*
Au_3_In	*a* = 5.907 *b* = 4.763 *c* = 5.210 (*a* = 5.857 *b* = 4.735 *c* = 5.150) [34] (*a* = 5.864 *b* = 4.746 *c* = 5.168) [36]	146.57 (145.85) Exp. [34] (143.83) Exp. [36]	0.893	141.1	26.7	75.4	0.411	5.285	−3.183	−14.399
Au_3_In_2_	*a* = *b* = 4.635 *c* = 5.691 (*a* = *b* = 4.537 *c* = 5.659) [35] (*a* = *b* = 4.540 *c* = 5.660) [37]	105.89 (100.88) Exp. [35] (101.03) Exp. [37]	0.794	103.7	23.1	64.6	0.396	4.489	−3.119	−17.639

**Table 6:** The calculated elastic constants of Au_3_In and Au_3_In_2_ (GPa).

	*C*_11_	*C*_12_	*C*_13_	*C*_14_	*C*_22_	*C*_23_	*C*_24_	*C*_33_	*C*_44_	*C*_55_	*C*_56_	*C*_66_
Au_3_In	160.0	135.2	121.7	-	192.7	119.7	-	173.1	28.7	31.5	-	26.8
Au_3_In_2_	134.8	95.8	75.9	−13.5	134.8	75.9	13.5	169.5	28.9	28.9	−13.5	19.5

**Table 7:** Calculated Vickers hardness *H*_v_ (GPa), fracture toughness *K*_IC_ (MPa·m^1/2^), plasticity index *δ*, universal anisotropy index *A^U^*, equivalent Zener anisotropy index *A*^eq^, universal log-Euclidean anisotropy index *A^L^*, percentage of elastic anisotropy in both compressibility *A^K^* (%) and shear *A^G^* (%), shear anisotropic factors *A*_1_, *A*_2_, and *A*_3_ of Au_3_In and Au_3_In_2_.

	*H_v_*	*K_IC_*	*δ*	*A^U^*	*A* *^eq^*	*A^L^*	*A* *^K^*	*A* *^G^*	*A*_1_	*A*_2_	*A*_3_
Au_3_In	1.76	0.999	4.92	0.24	1.56	0.10	0.51	2.26	1.28	0.99	1.30
Au_3_In_2_	1.54 (1.83) [17]	0.815	3.76	2.83	4.12	1.00	0.09	22.03	0.76	0.76	1.00

**Table 8:** The values of *E_max_*, *E_min_*, *G_max_*, *G_min_*, *v_max_*, *v_min_*, and the ratios of *E_max_*/*E_min_*, *G_max_*/*G_min_* and *v_max_*/*v_min_* for Au_3_In and Au_3_In_2_.

	*E* (GPa)			*G* (GPa)			*v*		
	*E_max_*	*E_min_*	Ratio	*G_max_*	*G_min_*	Ratio	*v_max_*	*v_min_*	Ratio
Au_3_In	85.88	50.69	1.69	31.52	19.45	1.62	0.69	0.22	3.19
Au_3_In_2_	119.65	30.10	3.98	41.27	9.86	4.18	0.92	−0.16	5.75

**Table 9:** Calculated density *ρ* (Kg/m^3^), sound velocities *V_l_*, *V_t_*, and *V_m_* (m/s), Debye temperature *Θ_D_* (K), thermal expansion coefficient *α* (1/K), volumetric heat capacity *ρC_p_* (J/m^3^K), wavelength of the dominant phonon at 300 K *λ_dom_* (m), minimum thermal conductivity *K_min_* (W/mK), and lattice thermal conductivity *K_ph_* (W/mK) of Au_3_In and Au_3_In_2_.

	*Θ_D_*	*α* (1 × 10^−6^)	*T_m_*	*ρC_P_* (1 × 10^6^)	*λ_dom_* (1 × 10^−12^)	$k_{\min}^{Clarke}$	$k_{\min}^{Cahill}$	$k_{ph}$
Au_3_In	165.3	48.6	1093.6	2.26	61.4	0.38	0.47	0.72
Au_3_In_2_	157.9	56.2	1012.7	1.96	56.4	0.35	0.43	0.60

## Data Availability

The data presented in this study are available upon reasonable request from the corresponding author due to confidentiality agreements.
